# Assessment of antibody responses to *Anopheles* SG6-P1 and *Aedes* N-term 34 kDa salivary peptides: a randomised human-challenge trial of controlled exposures to vector bites

**DOI:** 10.1186/s12916-026-04732-z

**Published:** 2026-03-02

**Authors:** Victor Chaumeau, Ellen A. Kearney, Praphan Wasisakun, Sunisa Sawasdichai, Aye Aye Aung, Paul A. Agius, Thaw Htwe Min, Daniela da Silva Gonçalves, Katherine O’Flaherty, Angeline Rouers, Sarang Aryalamloed, Gay Nay Htoo, Mu Phang Sue, Naw Moo Tha, Naw Chanida, Gornpan Gornsawun, Sadudee Chotirat, Julie A. Simpson, Laurent Rénia, François Nosten, Freya J. I. Fowkes

**Affiliations:** 1 Shoklo Malaria Research Unit, Mahidol Oxford Tropical Medicine Research Unit, Faculty of Tropical Medicine, Mahidol University, Mae Ramat, Tak Thailand; 2 Nuffield Department of Medicine, Centre for Tropical Medicine and Global Health, University of Oxford, Oxford, England UK; 3 Centre for Epidemiology and Biostatistics, Melbourne School of Population and Global Health, University of Melbourne, Melbourne, Australia; 4 Disease Elimination Program, Burnet Institute, Melbourne, Australia; 5 Department of Epidemiology and Preventive Medicine, Monash University, Melbourne, Australia; 6 Faculty of Health, Deakin University, Burwood, Australia; 7 A*STAR Infectious Diseases Labs, Agency for Science, Technology and Research, Singapore, Singapore; 8 Malaria Vivax Research Unit, Faculty of Tropical Medicine, Mahidol University, Bangkok, 10400 Thailand; 9 Lee Kong Chian School of Medicine, Nanyang Technological University, Singapore, Singapore; 10 School of Biological Sciences, Nanyang Technological University, Singapore, Singapore

**Keywords:** *Anopheles*, *Aedes*, Malaria, Dengue, Vector, Salivary antigen, Antibody biomarker, Serosurveillance, Human challenge, Southeast Asia

## Abstract

**Background:**

Human antibodies against mosquito salivary proteins are proposed as proxy biomarkers of exposure to vector bites. This trial sought to characterise the boosting and decay dynamics of antibodies against *Anopheles* SG6-P1 and *Aedes* N-term 34kDa salivary peptides in a human challenge model of controlled exposure to the main Southeast Asian malaria and global dengue vectors.

**Methods:**

In this single-centre, open-label, randomised, exploratory factorial trial, healthy volunteers aged 18–60 years with no history of recent travel to rural areas were recruited in Mae Sot, Thailand (ClincalTrials.gov: NCT04478370). Participants were randomly assigned to receive either 35 or 305 bites of mosquitos of laboratory-adapted colonies of *Anopheles dirus*, *Anopheles maculatus*, *Anopheles minimus*, *Aedes aegypti* and *Aedes albopictus* using a block randomisation schedule. Samples were collected weekly before, during and after the challenges for 16 weeks. The primary endpoint was total IgG antibodies against *Anopheles* SG6-P1 peptides measured using high-throughput ELISA and analysed with generalised estimating equations. Outcome assessors were masked to the intervention groups.

**Results:**

Between January 21, 2021, and May 10, 2022, 248 volunteers were screened, of whom 210 were randomly assigned to receive either 35 or 305 bites of *Ae. aegypti* (*n* = 20 and *n* = 19, respectively), *Ae. albopictus* (*n* = 20, *n* = 21), *An. dirus* (*n* = 21, *n* = 21), *An. maculatus* (*n* = 23, *n* = 24), or *An. minimus* (*n* = 22, *n* = 19), comprising the intention-to-treat population. In participants exposed to 305 *An. minimus* bites*,* total anti-gSG6-P1 IgG levels increased 1.14-fold (95% confidence interval [CI] 1.03–1.26) and 1.18-fold (95% CI 1.05–1.33) during the exposure and post-exposure periods respectively (relative to baseline), with minimal or no boosting observed in other groups. The estimated half-life of anti-gSG6-P1 antibodies was 421 (95% CI 155–688) days. Seven participants were withdrawn due to an adverse event.

**Conclusions:**

Anti-gSG6-P1 antibodies were boosted in response to exposure to 305 bites of *An. minimus*, but the magnitude of boosting was small and antibodies decayed slowly. Future research is warranted to identify and validate serological markers of vector biting exposures.

**Trial registration:**

The trial was registered in ClinicalTrials.gov: NCT04478370.

**Supplementary Information:**

The online version contains supplementary material available at 10.1186/s12916-026-04732-z.

## Background

Mosquitos are major vectors that cause approximately 350 million annual cases of important diseases, including malaria, dengue and chikungunya [1]. While there has been significant progress in reducing the burden of malaria in Southeast Asia in recent decades, increasing heterogeneity in transmission as cases decline is a barrier to achieving elimination. Surveillance of the vector populations has therefore been identified as a priority to monitor complex transmission dynamics and inform receptivity. The main vectors in this region are *Anopheles dirus*, *An. maculatus* and *An. minimus*; several other species are secondary vectors [2]. These species exhibit early and outdoor biting behaviours that limit the efficacy of mosquito bed nets [3]. Dengue viruses are transmitted by aedine mosquitos. *Aedes aegypti* and *Ae. albopictus* are the main vectors globally [4]. Infection can cause severe disease and death and the overall burden has increased over the past decades [5]. As there is no specific treatment and no safe, effective and widely available vaccine, prevention of infection with vector control and personal protection is critical to reduce morbidity and mortality.

Human exposure to vector bites is an important parameter of vectorial capacity and transmission models [6]. Measuring these exposures is therefore essential to assess the dynamics of disease transmission and evaluate the efficacy of vector control and transmission-blocking interventions. However, there is no tool to measure mosquito biting exposures accurately and at scale [7]. This knowledge gap constitutes an important barrier to disease control and elimination.

Upon blood feeding, female mosquitos inject their saliva into the skin of vertebrates. Mosquito saliva is composed of hundreds of biologically active components that play essential roles in the physiology of blood feeding and elicit immune responses in vertebrates [8, 9]. Assessment of antibodies directed against mosquito saliva in human blood samples is proposed as a surrogate measure of exposures to bites of medically important mosquito vector species [10]. Using omics approaches, dozens of *Anopheles*- and *Aedes*-specific salivary proteins have been discovered thereby opening avenues for the development of biomarkers of vector biting exposures [11, 12]. Initial investigations identified antibody responses to *Anopheles gambiae* salivary gland protein 6 (gSG6) and its peptide derivative (gSG6-P1) in individuals naturally bitten by *Anopheles* mosquitos in Africa [13, 14]. Similar investigations have identified the *Aedes aegypti* N-term 34 kDa (aeg34kDa) salivary peptide as a potential serological biomarker of exposure to *Aedes* bites [15]. Subsequently, measurement of human antibody responses to these salivary antigens has been used as a population-level outcome to assess vector biting exposures, estimate risk of disease transmission and evaluate vector control interventions globally [15–18]. To date, the leading candidate salivary antigens have primarily been investigated as biomarkers of species-specific biting exposure, e.g. gSG6 to measure *An. gambiae* exposure and aeg34kDa to measure *Ae. aegypti* exposure. Indeed, a systematic review with pooled analysis found that when applied in settings outside Africa where *An. gambiae* is absent, the association between human biting rates and anti-gSG6 IgG was weaker [16]. A study on La Reunion Island (where only *Ae. albopictus* is present) identified a positive association with antibodies against aeg34kDa and biting exposure [17]. This highlights a need for further investigations to quantify the effect of biting exposures from other *Anopheles* and *Aedes* species on the anti-gSG6 and anti-aeg34kDa antibody response. Additionally, this suggests a potential role for novel peptide orthologs to serve as species-specific markers of exposure to bites of *Ae. albopictus* and *Anopheles* species that predominate in regions outside of Africa. A species-specific approach may help overcome limitations of varied sequence identity between the SG6 and N-term 34 kDa proteins of *An. gambiae* and *Ae. aegypti* with other major *Anopheles* and *Aedes* species, respectively [11, 12]. This approach is being trialled to identify novel non-SG6 salivary antigens in South America [19, 20], where the SG6 gene is absent from the dominant vectors (*Anopheles darlingi* and *Anopheles albimanus);* however, it is yet to be trialled in Southeast Asia where the sequence identity to gSG6 ranges from 52 to 78% across the dominant vectors [11].

There is a key knowledge gap surrounding individual-level boosting and decay dynamics of anti-salivary antibodies following exposure to mosquito bites which prevents their scale-up for these proposed applications. This gap is primarily due to inherent challenges in observing an individual’s total mosquito biting exposure over time in natural settings, which may be better simulated under conditions of controlled exposure. To our knowledge, only one study has sought to characterise the individual-level longitudinal antibody responses following controlled human exposure to approximately 100 *Culex quinquefasciatus* bites fortnightly for ten months [21]. IgG antibodies to *Cx. quinquefasciatus* salivary extracts were measured 4 weekly and were found to boost and decay rapidly before being sustained across the remainder of the study, potentially suggesting the development of a tolerance. However, further studies would be required to confirm these observations as the sample size for this study was only one participant who had no prior exposure to *Cx. quinquefasciatus* mosquito bites. An expanded version of this study design that captures rich data on the number of bites received and the antibody response measured at the individual level could be useful to characterise the dynamics and dose–response relationship of antibody response to *Anopheles* and *Aedes* salivary antigens following biting exposures.

The aim of this study was to assess the boosting and decay dynamics of total IgG responses directed against the *Anopheles* SG6-P1 and *Aedes* N-term 34 kDa salivary peptides in a human challenge model of controlled exposure to bites of *An. dirus*, *An. maculatus*, *An. minimus* (the main malaria vectors in the Greater Mekong Subregion), *Ae. aegypti* and *Ae. albopictus* (the main dengue vectors worldwide).

## Methods

### Study design

This study is an exploratory, factorial randomised controlled trial of exposure to mosquito bites with ten arms corresponding to different species (*An. minimus*, *An. maculatus*, *An. dirus*, *Ae. aegypti* and *Ae. albopictus*) and numbers of bites (35 or 305 bites in total split in seven weekly challenges over 6 weeks). The study was conducted at the Shoklo Malaria Research Unit in Mae Sot, Thailand. The protocol, participant information sheet, and informed consent form were approved by the Oxford Tropical Research Ethics Committee (reference 62–19), the Ethics Committee of the Faculty of Tropical Medicine, Mahidol University (reference TMEC 20–007), the Alfred Hospital Ethics Committee (reference 630/21) and the Tak Community Advisory Board (reference TCAB202003), a community-based committee assembling members of the communities in which the study was performed [22]. The trial was registered in ClinicalTrials.gov (NCT04478370), and the detailed protocol was published separately [23]. The trial was published according to CONSORT (Consolidated Standards of Reporting Trials) 2025 guidelines (see Additional file 1: CONSORT checklist).

### Participants

Participants were generally healthy individuals aged 18 to 60 years old as assessed by a medical doctor, of Thai, Burmese, or Karen ethnicity, living in Mae Sot city for the last 12 months and able to tolerate mosquito biting exposures. Exclusion criteria included individuals with a history of travel in a rural area (i.e. may be exposed to *Anopheles* bites) in the last 12 months, or plan to do so during the study, medication, or condition deemed to interfere with the outcome measure or increase the risk of an adverse reaction to the study procedures, and haemoglobin concentration less than 110 g/L of blood. Ethnicity was not part of the statistical analysis plan, and therefore it was not recorded. History of travel prior to the 12-month exclusion criteria and working in rural or urban areas was not documented. This was deemed not relevant to the study because this trial sought to identify biomarkers of recent vector biting exposures that could be used to assess transmission dynamics and efficacy of vector control interventions in populations with previous exposures. Pregnant and breastfeeding women were also excluded. Information on the study was spread through word of mouth by the study team to people who live in Mae Sot, and individuals interested in participating were invited to contact the study team. To reach the planned sample size, interested volunteers were asked to further spread the study information amongst their networks. Gender was self-reported by the participants, and every answer provided by them was recorded. All participants gave their written informed consent for participating in the study.

### Randomisation and masking

A block randomisation schedule was generated by a study investigator (VC) using the variables species (*An. minimus*, *An. maculatus*, *An. dirus*, *Ae. aegypti* and *Ae. albopictus*) and dose (35 or 305 bites in total), yielding an ordered list of 15 blocks with ten participants per block, each participant randomly assigned to one of the ten study arms (i.e. 15 participants per study arm). Participants who could not complete the study were replaced; hence, the actual sample size ranged from 19 to 24 participants per study arm (see Results section for more details). This study was exploratory, and the sample size was small. Therefore, randomisation was not stratified over participants’ characteristics (e.g. age, sex or occupation). An allocation sequence was implemented using individual, sealed and sequentially numbered envelopes. Following screening and eligibility assessment, participants were assigned to a study arm during visit two using the randomisation schedule. The allocation sequence was generated by a study investigator. At the beginning of the study, the study coordinator prepared a set of case report forms with pre-printed subject identification codes and attached the sealed envelope containing intervention allocation to the case report forms. Then, the study nurses assigned a subject identification code to participants by chronological order of enrolment in the study, and the envelope was opened during visit two. The personnel who enrolled the participants and assigned participants to the interventions had no access to the random allocation sequence. Allocation of intervention was masked to outcome assessors (laboratory personnel who processed the serum samples). In order to do so, allocation of intervention in study datasets that contain this information was masked until all antibody data were made available to study investigators and statisticians.

### Procedures

Following screening and eligibility assessment, participants were appointed to attend weekly visits for 16 weeks and the complete follow-up was 112 days. Of note, there was some flexibility in the schedule to encourage participant retention—with 90% of sample collection occurring in 7 ± 3 days. Participants were challenged with bites of laboratory-adapted colonies of *An. minimus*, *An. maculatus*, *An. dirus*, *Ae. aegypti* or *Ae. albopictus* weekly between day 14 and day 56 (seven times in total). Participants in the low-exposure arms were challenged on each occasion with five mosquito bites (35 bites in total); those in the high-exposure arms were challenged once with five bites and then six times with 50 bites (305 bites in total). The decision to challenge every participant with five bites at the first challenge (including those in the high-exposure groups) was made to detect participants with hypersensitivity to mosquito bites and withdraw them from the study before the second challenge, so to ensure trial safety. The target number of mosquitos was put in a feeding cup (plastic container covered with netting fabric). The cup was attached to the participant’s leg and left undisturbed for 15 min. After 15 min, the cup was removed and the number of fed mosquitos was recorded. If not all mosquitos fed, the operation was repeated until the target number of fed mosquitos was obtained. The exact number of fed mosquitos was recorded for every challenge. All challenges were performed with 5- to 7-day-old starved nulliparous female imagoes (i.e. that had never blood fed before) of laboratory-adapted mosquito colonies reared in insectaries. Immediate skin reactions were recorded 20 to 30 min after every challenge and delayed skin reactions were recorded 24 to 36 h after the first and second challenges. The level of antibody titres against mosquito salivary antigens was measured in participant serum from venous blood collected at one-week intervals for the entire follow-up.

### Immunological assays

The peptide panel tested in this study included the gSG6-P1 peptide (derived from the SG6 protein sequence of *An. gambiae* and originally designed by Poinsignon et al. [14]), the corresponding orthologous peptides dirSG6-P1, macSG6-P1 and min-SG6-P1 (designed for this study using the SG6 protein sequence of *An. dirus*, *An. maculatus* and *An. minimus* published previously [11]), the N-term 34kDa peptide of *Ae. aegypti* (originally designed by Elanga Ndille et al. [15]) and the corresponding orthologous peptide in *Ae. albopictus* also designed in-house (the details of peptide design and sequences are provided in Additional file 2: Figure S1). Total IgG antibodies against these peptides were measured by adapting previously published ELISA protocols [14, 17, 24, 25], and optimising them into a high-throughput platform (detailed in Additional file 2: Supplementary Methodology). Antibody response is reported as levels (measured as optical density [OD]) and seropositivity (defined for each peptide as having an OD greater than the mean plus three standard deviations of the unexposed Melbourne controls [*n* = 8 from anonymised donors from the Red Cross in Melbourne, Australia]). Of note, *Anopheles minimus, An. maculatus, An. dirus, Aedes aegypti* and *Ae. albopictus* are all absent species from Melbourne, Australia; however, some other anopheline and aedine species do exist.

### Outcomes

The primary outcome of this study was the total IgG antibody responses to the *Anopheles* SG6-P1 salivary peptides. The secondary outcome was the total IgG antibody responses to the *Aedes* N-term 34kDa salivary peptides. Adverse events were assessed at every visit. Prespecified adverse events that would have required withdrawing the participant from the study included skin reactions greater than 30 mm in diameter, ecchymosis, vesicle, blister, bullae, Skeeter syndrome and systemic symptoms (generalised urticaria, angioedema and anaphylaxis).

### Statistical analysis

The change in individual antibody responses (as a continuous outcome and binary response) over time, and in response to mosquito biting exposures, was estimated according to the protocol using generalised linear mixed-effects models. Given we observed high levels of serial correlation in antibodies within individuals over time, generalised estimating equations (GEE) with robust standard errors were fitted to the longitudinal antibody data to account for the correlation within individuals and provide robust inference of the estimated effects of the exposure and post-exposure periods on antibody levels, relative to baseline.

The analysis estimates the change in mean antibody levels (log_2_ transformed) associated with the intervention (dummy indicators for biting exposure and post-exposure periods compared to the baseline), includes a linear term for time to measure per day changes in antibodies independent of the biting interventions and is adjusted for age and sex. The potential effect modification of the intervention group was explored by estimating interaction terms between the intervention period and intervention group (i.e. mosquito biting species and dose). Given we observed waning correlation in within-individual antibody levels over time, a 1 st order autoregressive working correlation structure was used in the GEE analyses to provide correct inference given the dependencies in the data (repeated antibody measurements).

The half-lives (*t*_*1/2*_) of each anti-salivary antibody were calculated using the estimates of change with time (per day, assumed linear slope) for the post-exposure period (for models that interact the intervention with time). As the antibody outcomes have been log2 transformed, the negative inverse of the estimated slopes were calculated to represent the number of days needed to achieve a 50% relative reduction in antibody levels. The statistical analyses were performed using STATA v18 and plots were generated in R.

### Role of the funding source

The funder of the study had no role in study design, data collection, data analysis, data interpretation or writing of the report.

## Results

**Fig. 1:**
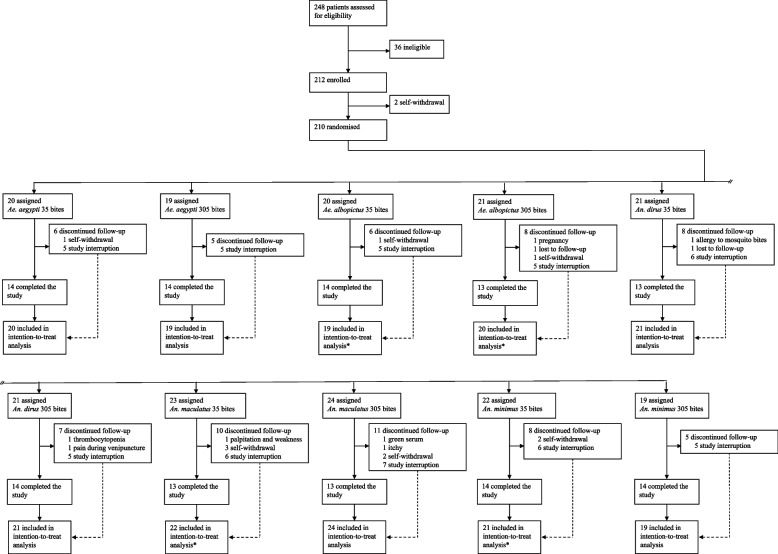
Flow diagram of trial design. *Participants contributing to only 1 sample were excluded from the analysis

Between January 21, 2021, and May 10, 2022, 248 volunteers were screened for eligibility, 212 were enrolled, 210 contributed at least one sample and 204 were challenged at least once with mosquito bites (Fig. 1). All participants who completed the challenge visits received the intended number of bites except for one participant in the *An. maculatus* low-dose group only received 25 out of 35 bites due to missing two visits. The study was interrupted on July 15, 2021, because of a COVID-19 outbreak at the study site and was resumed on November 3, 2021. At the time of study interruption, 55 participants had not completed the study; eleven participants who had attended 19/20 visits were not replaced, and 44 participants who had attended 17 visits or less were replaced with new participants when the study resumed (hence the deviation from expected sample size of 15 participants per study arm across 10 study arms). A total of seven participants had incomplete follow-up because of an adverse event (detailed in Additional file 2: Table S1). All participant data, excluding four individuals who only contributed one sample and were not challenged with mosquito bites, were included in the intention-to-treat analysis (*n* = 206, Fig. 1). Only 18 participants reported traveling to rural areas outside Mae Sot city during the study. All participants travelled only once, the median duration of overnight stay was four nights (IQR 2–5.5, range 0–15), only four (22%) travelled during the rainy season and 14 participants (78%) reported using personal protection with mosquito-bed nets and/or skin repellents during those trips.

**Fig. 2:**
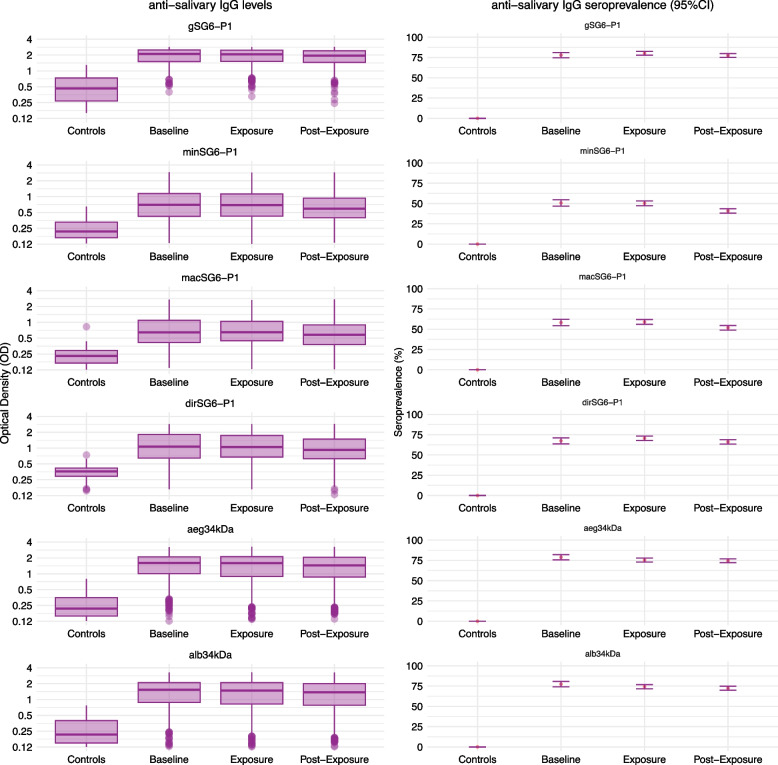
Levels and seroprevalence of antibodies against species-specific *Anopheles* SG6-P1 and *Aedes* N-term 34kDa salivary antigens in *n* = 210 participants at baseline, exposure and post-exposure periods, compared to unexposed Melbourne controls. Left panel shows box plots with median and interquartile range (IQR) of antibody levels (Optical Density), and right panel shows the seroprevalence and 95% confidence intervals (CI) for each of the salivary antigens. Sample sizes for the intervention periods are as follows: *n* = 620 baseline samples from 210 participants, *n* = 1134 exposure-period samples from 203 participants (one participant received bites but did not provide a sample), *n* = 1257 post-exposure samples from 176 individuals (see Additional file 2: Table S1 for details)

Participants’ baseline characteristics are shown in Table 1. Overall, 115 (54%) participants were male, and the median age was 30 years (inter-quartile range [IQR] 25–39.2). Participants had relatively high levels and seroprevalence of antibodies against *Anopheles* and *Aedes* salivary antigens (ranging from 50 to 78% across antigens; Additional file 2: Table S2) at baseline (prior to any challenge) compared to unexposed participants from Melbourne, Australia, who had low levels of antibodies and were all seronegative (Fig. 2). Baseline antibody levels were also found to vary according to enrolment date, with higher levels of antibodies observed prior to the interruption of the study (February to June 2021 compared to November 2021 to May 2022) (Additional file 2: Figure S2).

**Table 1:** Baseline characteristics of the cohort

	**AEG_35 (*n* = 20)**	**AEG_305 (*n* = 19)**	**ALB_35 (*n* = 20)**	**ALB_305 (*n* = 21)**	**DIR_35 (*n* = 21)**	**DIR_305 (*n* = 21)**	**MAC_35 (*n* = 23)**	**MAC_305 (*n* = 24)**	**MIN_35 (*n* = 22)**	**MIN_305 (*n* = 19)**
**Sex**										
*Male*	12 (60%)	10 (53%)	9 (45%)	9 (43%)	8 (38%)	15 (71%)	12 (52%)	16 (67%)	14 (64%)	9 (47%)
*Female*	8 (40%)	9 (47%)	11 (55%)	12 (57%)	13 (62%)	6 (29%)	11 (48%)	8 (33%)	8 (36%)	10 (53%)
**Age (years)**	31.5 (25–41)	30 (27–34.5)	31.5 (26.2–37.2)	29 (27–43)	28 (24–32)	31 (24–47)	26 (22–33)	33.5 (27.8–44.2)	35.5 (25.5–39.8)	30 (26–37)
**gSG6-P1**										
*Levels (OD)*	2.3 (1.54–2.51)	2.11 (1.66–2.44)	2.14 (1.5–2.43)	2.05 (1.43–2.49)	2.14 (1.48–2.47)	2.26 (1.75–2.49)	1.99 (1.58–2.51)	2.14 (1.39–2.48)	1.96 (1.59–2·5)	1.83 (1.29–2.46)
*Seroprevalence*	46/59 (78%)	46/57 (80.7%)	46/58 (79.3%)	46/61 (75.4%)	50/63 (79.4%)	52/62 (83.9%)	55/67 (82.1%)	53/72 (73.6%)	51/64 (79.7%)	38/57 (66.7%)
**minSG6-P1**										
*Levels (OD)*	0.84 (0.4–1.34)	0.72 (0.51–1.15)	0.71 (0.44–1.07)	0.69 (0.44–1.08)	0.67 (0.37–1.34)	0.75 (0.49–1.19)	0.71 (0.4–1.08)	0.65 (0.42–0.97)	0.64 (0.4–1.02)	0.55 (0.33–1.03)
* Seroprevalence*	33/59 (55.9%)	31/57 (54.4%)	31/58 (53.4%)	31/61 (50.8%)	31/63 (49.2%)	35/62 (56.5%)	36/67 (53.7%)	34/72 (47.2%)	31/64 (48.4%)	21/57 (36.8%)
**macSG6-P1**										
*Levels (OD)*	0.81 (0.5–1.21)	0.64 (0.43–0.85)	0.6 (0.4–0.94)	0.59 (0.4–1.16)	0.67 (0.47–1.43)	0.78 (0.43–1.07)	0.67 (0.47–1.22)	0.57 (0.45–1.07)	0.57 (0.4–1.01)	0.53 (0.38–0.86)
*Seroprevalence*	41/59 (69.5%)	35/57 (61.4%)	31/58 (53.4%)	31/61 (50.8%)	41/63 (65.1%)	40/62 (64.5%)	41/67 (61.2%)	40/72 (55.6%)	34/64 (53.1%)	27/57 (47.4%)
**dirSG6-P1**										
*Levels (OD)*	1.3 (0.63–1.79)	1.07 (0.72–1.51)	0.96 (0·63–1·51)	1.11 (0.68–1.97)	1.12 (0.63–1.97)	1.16 (0.69–2.13)	1.05 (0.72–1.73)	0.98 (0.64–1.66)	0.99 (0.61–1.77)	0.91 (0.56–1.54)
*Seroprevalence*	40/59 (67.8%)	42/57 (73.7%)	35/58 (60.3%)	43/61 (70.5%)	44/63 (69.8%)	44/62 (71%)	50/67 (74.6%)	45/72 (62.5%)	43/64 (67.2%)	32/57 (56.1%)
**aeg34kDa**										
*Levels (OD)*	1.61 (1.06–2.13)	1.64 (1.22–2.12)	1.43 (0.9–1.97)	1.65 (0.88–2.08)	1.88 (1.15–2.18)	1.70 (1.1–2.05)	1.47 (1.05–2.1)	1.73 (0.96–2.12)	1.55 (0.82–2.07)	1.51 (1.12–2.03)
*Seroprevalence*	45/59 (76.3%)	46/57 (80.7%)	45/58 (77.6%)	46/61 (75.4%)	54/63 (85·7%)	50/62 (80.6%)	57/67 (85.1%)	54/72 (75%)	45/64 (70.3%)	47/57 (82.5%)
**alb34kDa**										
*Levels (OD)*	1.56 (0.84–2.19)	1.5 (0.87–2.12)	1.28 (0.88–1.95)	1.52 (0.86–2.19)	1.76 (1.03–2.18)	1.68 (1.02–2.02)	1.35 (0.89–2.06)	1.65 (0.91–2.02)	1.38 (0.77–2.1)	1.45 (0.95–1.97)
*Seroprevalence*	44/59 (74.6%)	43/57 (75.4%)	46/58 (79.3%)	46/61 (75.4%)	52/63 (82.5%)	49/62 (79%)	55/67 (82.1%)	55/72 (76.4%)	46/64 (71.9%)	44/57 (77.2%)

Data are *n* (%), median (IQR), or *n*/*N* (%) for all randomised participants (*n* = 210). Antibody levels and seroprevalence were calculated using the 3 baseline samples collated by participant. *OD* optical density units

**Fig. 3:**
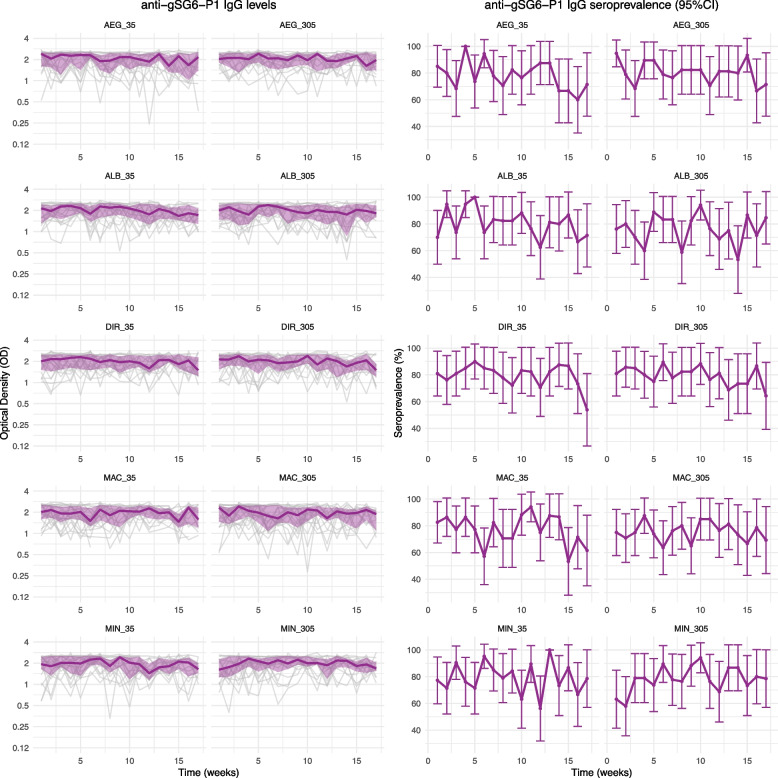
Observed levels and seroprevalence of antibodies against gSG6-P1 over time, by intervention group. Left panel shows spaghetti plots of the individual-level anti-gSG6-P1 IgG antibody response over time (week [days/7]), overlayed with the median and interquartile range of anti-gSG6-P1 antibody levels (OD). Right panel shows the seroprevalence and 95% confidence intervals (CI) each week. Panels represent each intervention group (biting species and dose), and arrows indicate weeks of biting exposure

**Fig. 4:**
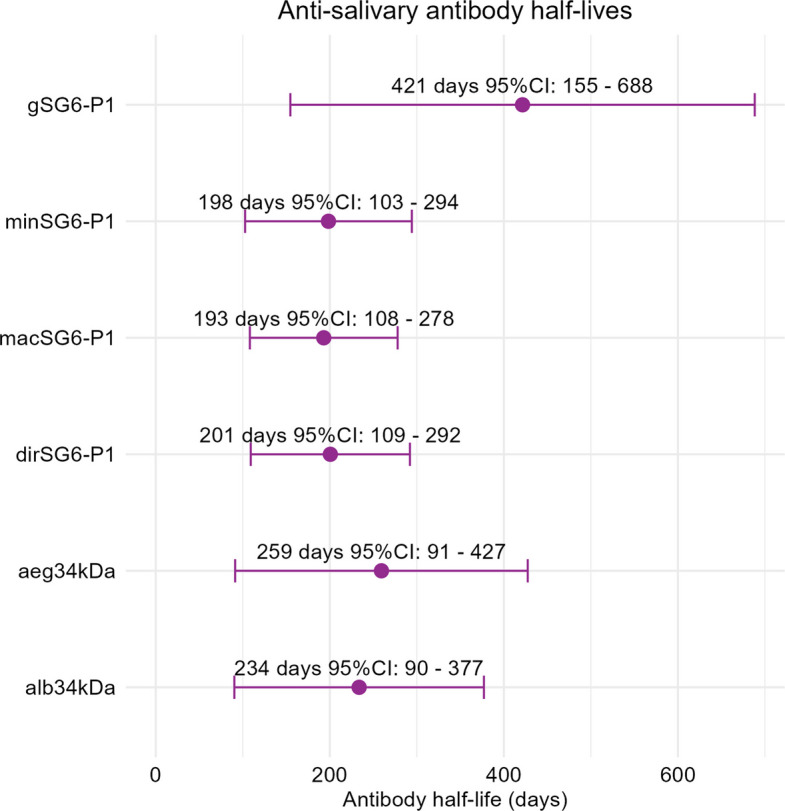
Estimated rates of antibody decay following biting exposure. Plot shows the half-lives (*t*_1/2_) and 95% confidence intervals (CI) for each anti-*Anopheles* and *Aedes* salivary antibody, estimated using linear slopes for the post-exposure period (for models that interact the intervention with time) and indicates the number of days needed for a 50% relative reduction in antibody levels. Model parameters were estimated using generalised estimating equations (GEEs) for *n* = 206 individuals with > 1 antibody measurement, with model output provided in Additional file 2: Table S4

Antibody responses against *Anopheles* salivary SG6-P1 antigens were highly dynamic over time, both within and between individuals (observed individual- and population-level responses by intervention group are presented in Fig. 3, and Additional file 2: Figures S3-S7). Analyses investigating the overall effect of mosquito biting exposure determined that IgG antibodies to *Anopheles* SG6-P1 salivary antigens decayed over time but were associated with boosts in antibody levels during the exposure and post-exposure periods (Additional file 2: Table S3). The magnitude and confidence intervals associated with these estimates varied according to the antigen investigated and are detailed in Additional file 2. The rate of antibody decay following biting exposure (i.e. half-life for the post-exposure period) was found to range from 193 (95% confidence interval [CI] 108–278) to 421 (95% CI 155–688) days depending on the SG6-P1 ortholog (Fig. 4; half-lives specific for species of exposure reported in Additional file 2: Figure S8).

**Fig. 5:**
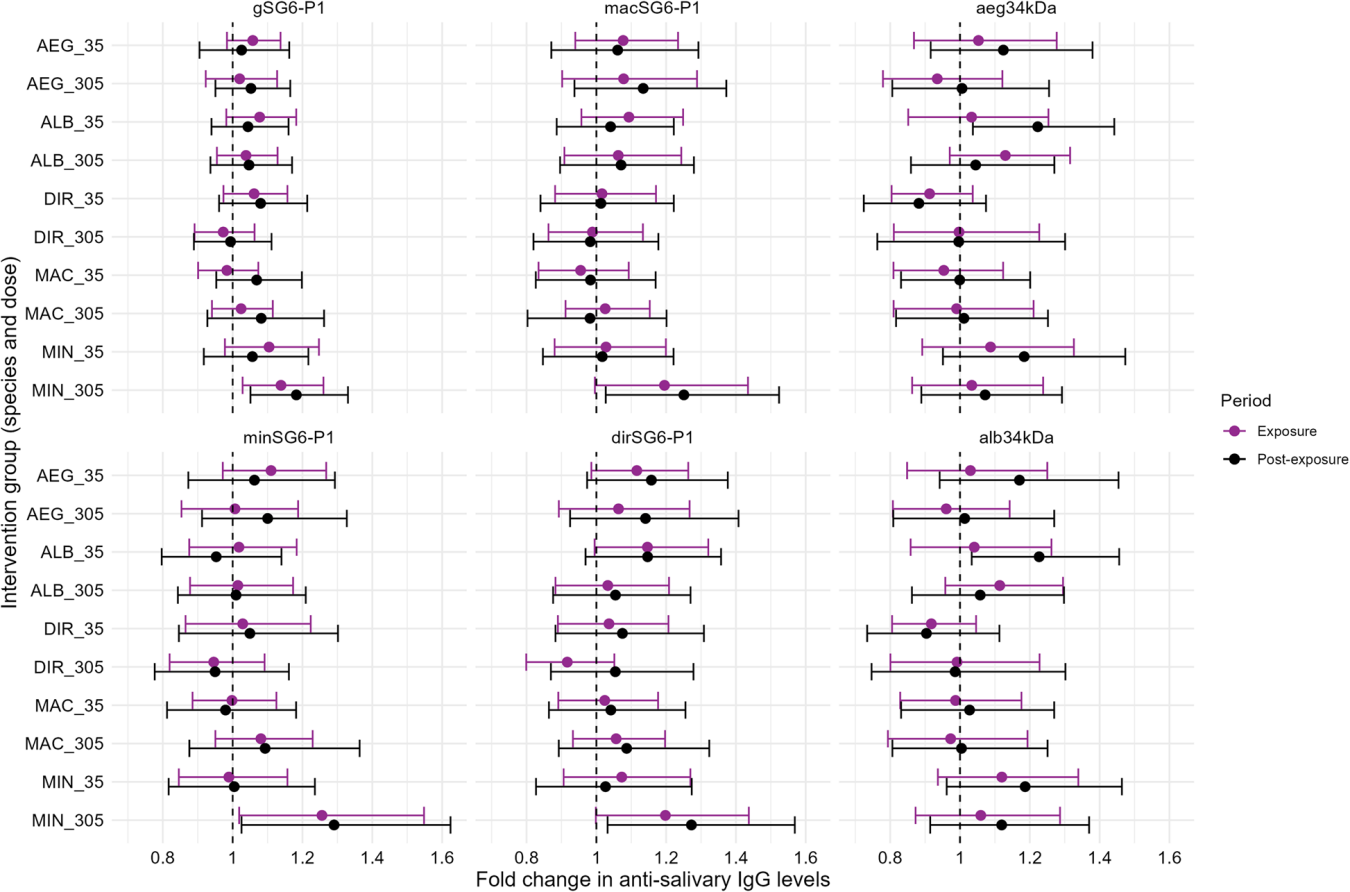
Effect of mosquito biting exposure, by intervention group (i.e. biting species and dose), on anti-salivary antibody levels. Forest plots represent the relative change in geometric mean antibody levels (95% confidence intervals) associated with the exposure and post-exposure periods compared to baseline for each intervention group (*Ae. aegypti* 35 bites, *Ae. aegypti* 305 bites, *Ae. albopictus* 35 bites, *Ae. albopictus* 305 bites, *An. dirus* 35 bites, *An. dirus* 305 bites, *An. maculatus* 35 bites, *An. maculatus* 305 bites, *An. minimus* 35 bites, *An. minimus* 305 bites). Model parameters were estimated using generalised estimating equations (GEEs) for *n* = 206 individuals with > 1 antibody measurements, with model output provided in Additional file 2: Table S5

**Fig. 6:**
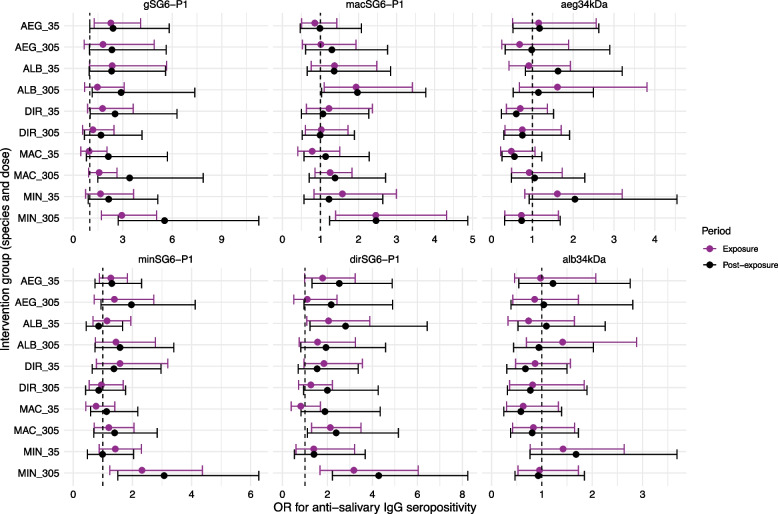
Effect of mosquito biting exposure, by intervention group (i.e. biting species and dose), on anti-salivary antibody seroprevalence. Forest plots represent the estimated change in the odds (i.e. odds ratio (OR), 95% Confidence Intervals) associated with the exposure and post-exposure periods compared to baseline for each intervention group (*Ae. aegypti* 35 bites, *Ae. aegypti* 305 bites, *Ae. albopictus* 35 bites, *Ae. albopictus* 305 bites, *An. diru*s 35 bites, *An. dirus* 305 bites, *An. maculatus* 35 bites, *An. maculatus* 305 bites, *An. minimus* 35 bites, *An. minimus* 305 bites). Model parameters were estimated using generalised estimating equations (GEEs) for *n* = 206 individuals with > 1 antibody measurements, with model output provided in Additional file 2: Table S6

The species- and dose-specific effects of mosquito biting exposure on the anti-salivary antibody response were determined. The largest boosts in antibody levels and seroprevalence during the exposure and post-exposure periods were observed amongst the individuals exposed to a high dose (305 bites over 6 weeks) of *An. minimus* bites. For example, anti-gSG6-P1 IgG antibody levels for the high-dose *An. minimus* group were boosted 1.14-fold (geometric mean ratio) (95% CI 1.03–1.26, *p* = 0.012) and 1.18-fold (95% CI 1.05–1.33, *p* = 0.005) during the exposure and post-exposure periods, respectively (relative to baseline), with minimal or no boosting in IgG levels observed in other groups (Fig. 5). Similarly, the largest increases in the odds of anti-gSG6-P1 IgG seropositivity during the exposure (odds ratio [OR] 2.94, 95% CI 2.71–5.05, *p* < 0.001) and post-exposure (OR 5.54, 95% CI 2.73–11.24, *p* < 0.001) periods were observed for the high-dose *An. minimus* intervention group (Fig. 6). Similar associations were observed across SG6-P1 orthologs.

Antibody responses to aeg34kDa and alb34kDa salivary antigens were also dynamic over time, and were found to have respective half-lives of 259 (95% CI 90–427) and 234 (95% CI 90–377) days following biting exposure (Fig. 4). The largest boosts in antibody levels were observed amongst the 35 *Ae. albopictus* biting group: in this group, anti-aeg34kDa and anti-alb34kDa IgG levels increased 1.22-fold (95% CI 1.04–1.44, *p* = 0.017) and 1.22-fold (95% CI 1.03–1.46, *p* = 0.019) in the post-exposure period relative to baseline, respectively (Fig. 5). Similar boosts were observed amongst those exposed to 35 bites of *Ae. aegypti;* however, the confidence intervals associated with these estimates were wide (aeg34kDa: 1.12-fold, 95% CI 0.92–1.38, *p* = 0.262; alb34kDa: 1.17, 95% CI 0.94–1.45, *p* = 0.156). Mosquito biting exposures were not associated with significant increases in anti-*Aedes* salivary antibody levels in the other intervention groups.

## Discussion

In this trial of controlled exposure to vector bites, total IgG responses to *Anopheles* SG6-P1 and *Aedes* N-term 34kDa salivary peptides were assessed in volunteers bitten by mosquitos of laboratory-adapted colonies of *An. dirus*, *An. maculatus*, *An. minimus* (the main malaria vectors in the Greater Mekong Subregion), *Ae. aegypti* and *Ae. albopictus* (the main dengue vectors worldwide). Most participants had detectable antibodies prior to mosquito biting challenges and total IgG responses to SG6-P1 peptides were boosted during and after exposures to 305 bites of *An. minimus* split in seven challenges over 6 weeks, but not after exposures to smaller numbers of bites or bites of other vector species. Similarly, total IgG antibody responses to 34kDa peptides were boosted following exposure to 35 total bites of *Ae. albopictus* but were not associated with significant boosts in antibody responses in the other groups. Overall, the increases in antibody titres were relatively small, and anti-salivary antibodies decayed slowly after cession of exposure.

This study identified that significant boosts in antibodies against gSG6-P1 were only observed for the group exposed to a large number of bites from *An. minimus*, whose SG6-P1 peptide sequence shares the greatest identity to gSG6-P1 (87% [11]). This suggests that the level of identity of the SG6 ortholog (injected during bite exposure from a non-*An. gambiae* species) to gSG6-P1 may be important in determining the degree of antibody cross-reactivity. This has important implications for the use of gSG6 outside of Africa, as low cross-reactivity may reduce its sensitivity and limit its application as a genus-level biomarker of exposure to the bites of all relevant *Anopheles* mosquito species. Antibody responses to the SG6-P1 orthologs were similar over time and in response to mosquito biting exposures, with boosting upon biting exposure consistently identified only amongst the *An. minimus* high-exposure arm (305 bites per person). The weaker non-significant boost in antibody levels across the SG6-P1 antigens for the *An. maculatus* and *An. dirus* exposure groups may be a result of their lower sequence identity to gSG6-P1 (78% and 48% respectively [11]), potentially resulting in less immunogenic epitopes from the P1 region being present in these novel antigens. Additional novel *Anopheles* salivary antigens corresponding to alternate regions of the SG6 protein (which bioinformatic analysis has predicted higher immunogenicity in the terminal region, Additional file 2: Figure S1) or one of dozens of other identified *Anopheles*-specific salivary proteins [11] could readily be investigated and validated as sensitive biomarkers of species-specific exposure in this cohort.

Study participants were found to have relatively high (yet variable) levels of anti-SG6-P1 and anti-34kDa antibodies at baseline, particularly compared to unexposed controls from Melbourne, Australia. This suggests that these antibodies recognise prior biting exposure to bites of *Anopheles* and *Aedes* mosquitos. These baseline antibody responses were ideal to mimic people naturally exposed to vector bites in endemic areas, as are the participants enrolled in trials of vector control and serosurveillance studies. As we excluded participants who had travelled to rural areas in the past 12 months (and the randomised study design should account for differences due to travel during the study), this finding is consistent with the slow decay rate of anti-salivary antibodies reported in this assessment. The anti-gSG6-P1 IgG half-life estimate reported in this assessment is comparable to the only other published estimate (424 days [26] which was determined without detailed knowledge of biting exposure but instead time since malaria infection). These long antibody half-lives challenge previous claims that total IgG responses to SG6-P1 peptides decay rapidly following interrupted exposure to mosquito bites [27]. This result has important implications for how these anti-salivary antibodies can be used in future studies. For example, they may be better suited to investigating longer-term changes in transmission dynamics, and studies employing them as outcomes in vector control trials will need to allow for sufficient follow-up to evaluate the delayed effects of the intervention so that its effectiveness is not underestimated due to the persistence of antibodies after biting exposure is reduced. As different antibody isotypes and subclasses have more dynamic responses than others [28], additional work is on-going to characterise their distinct longitudinal profiles within this cohort.

The high levels and seroprevalence of IgG antibodies at baseline (prior to controlled biting) may have contributed to the relatively small magnitude of antibody boosting associated with biting exposure observed in this trial, suggesting the development of an immune tolerance after repeated biting exposures. Evidence for this tolerance in previous studies is conflicted, reporting both positive and negative associations between anti-salivary antibodies and age [13, 29]. Alternatively, minimal boosting may be a result of the relatively small antigenic stimulus of mosquito salivary proteins injected during a bite and may suggest that individuals may need to be exposed to greater numbers of bites to register larger boosts in antibody levels. As some entomological surveys from the Greater Mekong Subregion report biting rates ranging between 184 and 694 bites per person per month [3], these salivary antibody biomarkers may still be applicable to quantify variation in biting rates throughout the region. Additional investigations of the sensitivity of these antibodies to detect differing levels of biting exposures are warranted.

Due to the complexity of quantifying an individual’s natural mosquito biting exposures, this trial uses a human challenge framework to control participants’ biting exposures and characterise the longitudinal dynamics of the anti-salivary antibody responses. This novel approach is a key strength of this study, which provides a valuable framework for future research striving to develop biomarkers of recent exposures to vector bites. However, there are several limitations to acknowledge. We used a factorial design because the trial was exploratory and the sample size per group was small. Therefore, the study was not powered to detect small-sized effects. Due to the ubiquitous nature of *Aedes* mosquitos, unobserved exposures to *Aedes* bites not related to the study challenges are likely to have occurred, potentially confounding the estimation of anti-34kDa antibody boosting and decay rates. While we attempted to control for this by providing participants with topical repellent, repellent use was not directly observed or reported. The significant variation in the baseline antibody responses observed across the enrolment dates was difficult to explain but may have been caused by seasonality, past history of exposure to *Anopheles* bites or travel restrictions during the COVID-19 pandemic. Moreover, baseline IgG responses to SG6-P1 peptides were lower in the groups of participants challenged with 305 bites of *An. minimus* which may have confounded the estimated effect of biting exposure on antibody levels within this group. We used 5- to 7-day-old nulliparous mosquitos of laboratory-adapted colonies, and the expression of SG6 was not characterised. Previous studies have reported variation of mosquito salivary proteome with mosquito age [30], infection [31] and resistance [32]. Hence, the proteome of the mosquitos used in this study may be different from that of wild mosquitos biting humans in natural settings. Although a more detailed characterisation of salivary gland proteome was out of the scope of this study, this should be the focus of future research. Only SG6-P1 and N-term 34kDa peptides were tested. Although synthetic peptides are useful for high-throughput screening at scale, they do not capture conformational epitopes and epitopes of other types of biomolecules (e.g. glycoproteins) that may be important targets of antibody responses to mosquito salivary components.

Future research should strive to screen additional targets, including whole saliva protein extract, salivary proteins in their native form, and synthetic peptides. Mosquito species diversity is particularly high in this region, and many relevant vector species were not assessed. Colonisation of mosquitos is challenging, and many vectors have not been colonised yet. Additional research striving to establish colonies for relevant mosquito vector species should be encouraged for inclusion in future trials of controlled mosquito biting exposure. Furthermore, this vector salivary biomarker approach could readily be extended to other medically important arthropods and combined with pathogen serology as an interesting approach to surveillance of vector-borne diseases, but additional research is needed before these tools can be implemented programmatically [25].

## Conclusions

This study demonstrated small boosts in antibody responses directed against mosquito salivary antigens following controlled exposure to mosquito bites in a population with high levels of antibodies during baseline. Additional effort is needed to develop reliable biomarkers of recent exposures to vector bites.

## Supplementary Information

Additional file 1: Trial CONSORT checklist.

Additional file 2: Contains Additional file 2: Supplementary methodology, Additional file 2: Figures S1-S8 and Additional file 2: Tables S1-S7 associated with the manuscript. FigS1: Alignment of SG6 and 34kDa salivary protein sequences across species. FigS2: Anti-salivary antibody data at first baseline visit by enrolment month. FigS3: Observed levels and seroprevalence of antibodies against minSG6-P1 over time, by intervention group. FigS4: Observed levels and seroprevalence of antibodies against macSG6-P1 over time, by intervention group. FigS5: Observed levels and seroprevalence of antibodies against dirSG6-P1 over time, by intervention group. FigS6: Observed levels and seroprevalence of antibodies against aeg34kDa over time, by intervention group. FigS7: Observed levels and seroprevalence of antibodies against alb34kDa over time, by intervention group. FigS8. Estimated rates of antibody decay following species-specific biting exposure. Table S1: Sample schedule including any adverse events. Table S2: Observed values of total IgG antibody responses by intervention group and follow-up period. Table S3: Effect of mosquito biting exposure period (across all intervention groups) on anti-salivary antibody levels. Table S4: Time-dependent effect of mosquito biting exposure period (across all intervention groups) on boosting and decay of anti-salivary antibodies. Table S5: Effect of mosquito biting exposure period, modified by intervention group (species and dose), on anti-salivary antibody levels. Table S6: Effect of mosquito biting exposure, modified by intervention group (species and dose), on anti-salivary antibody seroprevalence. Table S7: Pairwise comparison of SG6-P1 sequences across SG6 orthologs in the Southeast Asian malaria vector species.

## Data Availability

The dataset and do file generated and analysed during the current study are available on Zenodo repository: https://doi.org/10.5281/zenodo.18529718 [33]. Please note, to comply with ethics regulations, we have removed any potentially identifiable data from the dataset (including variables: age, sex, date of visit, etc.). As models in the manuscript adjusted for age and sex, please note that final reported values may differ slightly.

## Abbreviations

aeg (prefix): *Aedes aegypti*
alb (prefix): *Aedes albopictus*
CI: Confidence interval
dir (prefix): *Anopheles dirus*
ELISA: Enzyme-linked immunosorbent assay
g (prefix): *Anopheles gambiae*
GEE: Generalised estimating equations
GMR: Geometric mean ratio
mac (prefix): *Anopheles maculatus*
min (prefix): *Anopheles minimus*
OD: Optical density
OR: Odds ratio
SG6: Salivary gland 6
SG6-P1: Salivary gland 6 peptide 1

## References

[1] World Health Organization. A global brief on vector-borne diseases. Geneva: World Health Organization; 2014. p. 54. https://iris.who.int/handle/10665/111008.

[2] Sinka ME, Bangs MJ, Manguin S, Chareonviriyaphap T, Patil AP, Temperley WH, et al. The dominant *Anopheles* vectors of human malaria in the Asia-Pacific region: occurrence data, distribution maps and bionomic précis. Parasitol Vectors. 2011;4:89. 10.1186/1756-3305-4-89. 10.1186/1756-3305-4-89 PMC3127851 21612587

[3] Chaumeau V, Fustec B, Nay Hsel S, Montazeau C, Naw Nyo S, Metaane S, et al. Entomological determinants of malaria transmission in Kayin state, Eastern Myanmar: A 24-month longitudinal study in four villages. Wellcome Open Res. 2018;3:109. 10.12688/wellcomeopenres.14761.4. 31206035 10.12688/wellcomeopenres.14761.1 PMC6544137

[4] Guzman MG, Gubler DJ, Izquierdo A, Martinez E, Halstead SB. Dengue infection. Nat Rev Dis Primers. 2016;2:16055. 27534439 10.1038/nrdp.2016.55

[5] Wartel TA, Prayitno A, Hadinegoro SRS, Capeding MR, Thisyakorn U, Tran NH, et al. Three decades of dengue surveillance in five highly endemic South East Asian countries: a descriptive review. Asia Pac J Public Health. 2017;29:7–16. 10.1177/1010539516675701. 28198645 10.1177/1010539516675701

[6] Brady OJ, Godfray HCJ, Tatem AJ, Gething PW, Cohen JM, McKenzie FE, et al. Vectorial capacity and vector control: reconsidering sensitivity to parameters for malaria elimination. Trans R Soc Trop Med Hyg. 2016;110:107–17. 26822603 10.1093/trstmh/trv113 PMC4731004

[7] Monroe A, Moore S, Okumu F, Kiware S, Lobo NF, Koenker H, et al. Methods and indicators for measuring patterns of human exposure to malaria vectors. Malar J. 2020;19:207. 10.1186/s12936-020-03271-z. 32546166 10.1186/s12936-020-03271-z PMC7296719

[8] Ribeiro JM, Francischetti IM. Role of arthropod saliva in blood feeding: sialome and post-sialome perspectives. Annu Rev Entomol. 2003;48:73–88. 12194906 10.1146/annurev.ento.48.060402.102812

[9] Guerrero D, Vo HTM, Lon C, Bohl JA, Nhik S, Chea S, et al. Evaluation of cutaneous immune response in a controlled human in vivo model of mosquito bites. Nat Commun. 2022;13:7036. 10.1038/s41467-022-34534-9. 36396947 10.1038/s41467-022-34534-9 PMC9672097

[10] Kearney EA, Heng-Chin AS, O’Flaherty K, Fowkes FJI. Human antibodies against Anopheles salivary proteins: emerging biomarkers of mosquito and malaria exposure. Trends Parasitol. 2025;41:361–73. 10.1016/j.pt.2025.03.009. 40246632 10.1016/j.pt.2025.03.009

[11] Arcà B, Lombardo F, Struchiner CJ, Ribeiro JM. Anopheline salivary protein genes and gene families: an evolutionary overview after the whole genome sequence of sixteen *Anopheles* species. BMC Genomics. 2017;18:153. 28193177 10.1186/s12864-017-3579-8 PMC5307786

[12] Arcà B, Lombardo F, Francischetti IMB, Pham VM, Mestres-Simon M, Andersen JF, et al. An insight into the sialome of the adult female mosquito *Aedes albopictus*. Insect Biochem Mol Biol. 2007;37:107–27. 10.1016/j.ibmb.2006.10.007. 17244540 10.1016/j.ibmb.2006.10.007

[13] Rizzo C, Ronca R, Fiorentino G, Verra F, Mangano V, Poinsignon A, et al. Humoral response to the *Anopheles gambiae* salivary protein gSG6: a serological indicator of exposure to Afrotropical malaria vectors. PLoS ONE. 2011;6:e17980. 10.1371/journal.pone.0017980. 21437289 10.1371/journal.pone.0017980 PMC3060095

[14] Poinsignon A, Cornelie S, Mestres-Simon M, Lanfrancotti A, Rossignol M, Boulanger D, et al. Novel peptide marker corresponding to salivary protein gSG6 potentially identifies exposure to *Anopheles* bites. PLoS ONE. 2008;3:e2472. 10.1371/journal.pone.0002472. 18575604 10.1371/journal.pone.0002472 PMC2427200

[15] Elanga Ndille E, Doucoure S, Damien G, Mouchet F, Drame PM, Cornelie S, et al. First attempt to validate human IgG antibody response to Nterm-34kDa salivary peptide as biomarker for evaluating exposure to *Aedes aegypti* bites. PLoS Negl Trop Dis. 2012;6:e1905. 10.1371/journal.pntd.0001905. 23166852 10.1371/journal.pntd.0001905 PMC3499371

[16] Kearney EA, Agius PA, Chaumeau V, Cutts JC, Simpson JA, Fowkes FJI. *Anopheles* salivary antigens as serological biomarkers of vector exposure and malaria transmission: a systematic review with multilevel modelling. Elife. 2021;10:e73080. 10.7554/eLife.73080. 34939933 10.7554/eLife.73080 PMC8860437

[17] Elanga Ndille E, Doucoure S, Poinsignon A, Mouchet F, Cornelie S, D’Ortenzio E, et al. Human IgG antibody response to *Aedes* Nterm-34kDa salivary peptide, an epidemiological tool to assess vector control in chikungunya and dengue transmission area. PLoS Negl Trop Dis. 2016;10:e0005109. 10.1371/journal.pntd.0005109. 27906987 10.1371/journal.pntd.0005109 PMC5131890

[18] Williams RJ, Swinehart BD, Abouneameh S, Rutha IJ, Msolo DC, Tarimo B, et al. Antibody responses to SG6, AgSAP, and SAMSP1 following Anopheline salivary exposure. medRxiv. 2025;:2025.07.14.25331506. 10.1101/2025.07.14.25331506. 10.1186/s13071-025-07072-8 PMC12560312 41146309

[19] Londono-Renteria B, Drame PM, Montiel J, Vasquez AM, Tobón-Castaño A, Taylor M, et al. Identification and pilot evaluation of salivary peptides from Anopheles albimanus as biomarkers for bite exposure and malaria infection in Colombia. Int J Mol Sci. 2020;21. 10.3390/ijms21030691. 10.3390/ijms21030691 PMC7037407 31973044

[20] Londono-Renteria B, Montiel J, Calvo E, Tobón-Castaño A, Valdivia HO, Escobedo-Vargas K, et al. Antibody responses against Anopheles darlingi immunogenic peptides in plasmodium infected humans. Front Cell Infect Microbiol. 2020;10. 10.3389/fcimb.2020.00455. 10.3389/fcimb.2020.00455 PMC7488213 32984076

[21] Peng Z, Simons FE. A prospective study of naturally acquired sensitization and subsequent desensitization to mosquito bites and concurrent antibody responses. J Allergy Clin Immunol. 1998;101(2 Pt 1):284–6. 10.1016/s0091-6749(98)70395-1. 9500765 10.1016/s0091-6749(98)70395-1

[22] Cheah PY, Lwin KM, Phaiphun L, Maelankiri L, Parker M, Day NP, et al. Community engagement on the Thai-Burmese border: rationale, experience and lessons learnt. Int Health. 2010;2:123–9. 10.1016/j.inhe.2010.02.001. 22984375 10.1016/j.inhe.2010.02.001 PMC3442337

[23] Sawasdichai S, Chaumeau V, Kearney E, Wasisakun P, Simpson JA, Price DJ, et al. Characterizing antibody responses to mosquito salivary antigens of the Southeast Asian vectors of malaria and dengue with a human challenge model of controlled exposure: a protocol. Wellcome Open Res. 2023;8:135. 10.12688/wellcomeopenres.19049.2. 37456919 10.12688/wellcomeopenres.19049.2 PMC10338987

[24] Montiel J, Carbal LF, Tobón-Castaño A, Vásquez GM, Fisher ML, Londono-Rentería B. IgG antibody response against *Anopheles* salivary gland proteins in asymptomatic *Plasmodium* infections in Narino, Colombia. Malar J. 2020;19:42. 10.1186/s12936-020-3128-9. 31973737 10.1186/s12936-020-3128-9 PMC6979332

[25] Kearney EA, Amratia P, Kang SY, Agius PA, Alene KA, O’Flaherty K, et al. Geospatial joint modeling of vector and parasite serology to microstratify malaria transmission. Proc Natl Acad Sci U S A. 2024;121:e2320898121. 10.1073/pnas.2320898121. 38833464 10.1073/pnas.2320898121 PMC11181033

[26] Kerkhof K, Sluydts V, Willen L, Kim S, Canier L, Heng S, et al. Serological markers to measure recent changes in malaria at population level in Cambodia. Malar J. 2016;15:529. 10.1186/s12936-016-1576-z. 27809852 10.1186/s12936-016-1576-z PMC5096337

[27] Drame PM, Poinsignon A, Besnard P, Cornelie S, Le Mire J, Toto J-C, et al. Human antibody responses to the *Anopheles* salivary gSG6-P1 peptide: a novel tool for evaluating the efficacy of ITNs in malaria vector control. PLoS ONE. 2010;5:e15596. 10.1371/journal.pone.0015596. 21179476 10.1371/journal.pone.0015596 PMC3001874

[28] Vidarsson G, Dekkers G, Rispens T. IgG subclasses and allotypes: from structure to effector functions. Front Immunol. 2014;5:520. 10.3389/fimmu.2014.00520. 25368619 10.3389/fimmu.2014.00520 PMC4202688

[29] Badu K, Siangla J, Larbi J, Lawson BW, Afrane Y, Ong’echa J, et al. Variation in exposure to *Anopheles gambiae* salivary gland peptide (gSG6-P1) across different malaria transmission settings in the western Kenya highlands. Malar J. 2012;11:318. 10.1186/1475-2875-11-318. 22963464 10.1186/1475-2875-11-318 PMC3541077

[30] Phattanawiboon B, Jariyapan N, Mano C, Roytrakul S, Paemanee A, Sor-Suwan S, et al. Salivary gland proteome during adult development and after blood feeding of female *Anopheles dissidens* mosquitoes (Diptera: Culicidae). PLoS ONE. 2016;11:e0163810. 10.1371/journal.pone.0163810. 27669021 10.1371/journal.pone.0163810 PMC5036837

[31] Marie A, Holzmuller P, Tchioffo MT, Rossignol M, Demettre E, Seveno M, et al. *Anopheles gambiae* salivary protein expression modulated by wild *Plasmodium falciparum* infection: highlighting of new antigenic peptides as candidates of *An. gambiae* bites. Parasit Vectors. 2014;7:599. 10.1186/s13071-014-0599-y. 25526764 10.1186/s13071-014-0599-y PMC4287575

[32] Omoke D, Impoinvil LM, Derilus D, Okeyo S, Saizonou H, Mulder N, et al. Whole transcriptomic analysis reveals overexpression of salivary gland and cuticular proteins genes in insecticide-resistant *Anopheles arabiensis* from Western Kenya. BMC Genomics. 2024;25:313. 10.1186/s12864-024-10182-9. 38532318 10.1186/s12864-024-10182-9 PMC10967204

[33] Kearney EA, Chaumeau V, Wasisakun P, Sawasdichai, Aung AA, Agius PA, et al. Assessment of antibody responses to Anopheles SG6-P1 and Aedes N-term 34kDa salivary peptides: a randomised human-challenge trial of controlled exposures to vector bites: data sets. (v1.0) . Zenodo. 10.5281/zenodo.18529718. 10.1186/s12916-026-04732-z PMC13059329 41765941

